# Supplementary material for: Quantitative in vivo bioluminescence imaging of orthotopic patient-derived glioblastoma xenografts
Source: Sci Rep. 2020 Sep 21;10:15361. doi: 10.1038/s41598-020-72322-x (PMC7506024; doi:10.1038/s41598-020-72322-x)
Supplement: Supplementary file 1 — Supplementary Figures. [file 41598_2020_72322_MOESM1_ESM.pdf]

Supplemental Information

Quantitative *in vivo* bioluminescence imaging of orthotopic patient-derived glioblastoma xenografts

Anna L. Koessinger, Dominik Koessinger, Katrina Stevenson, Catherine Cloix, Louise Mitchell, Colin Nixon, Natividad Gomez-Roman, Anthony J. Chalmers, Jim C. Norman, Stephen W.G. Tait

Supplemental Figure 1
Full length scans of blots shown in Figure 2

Supplemental Figure 2
FACS analysis of G1 GSC iRFP
Cell confluence assay after treatment with chemotherapeutic agents

Supplemental Figure 3
H&E and immunohistochemistry for Ki67 and SOX2 in E2 an G7 parental vs. iRFP orthotopic xenografts

Supplemental Figure 1

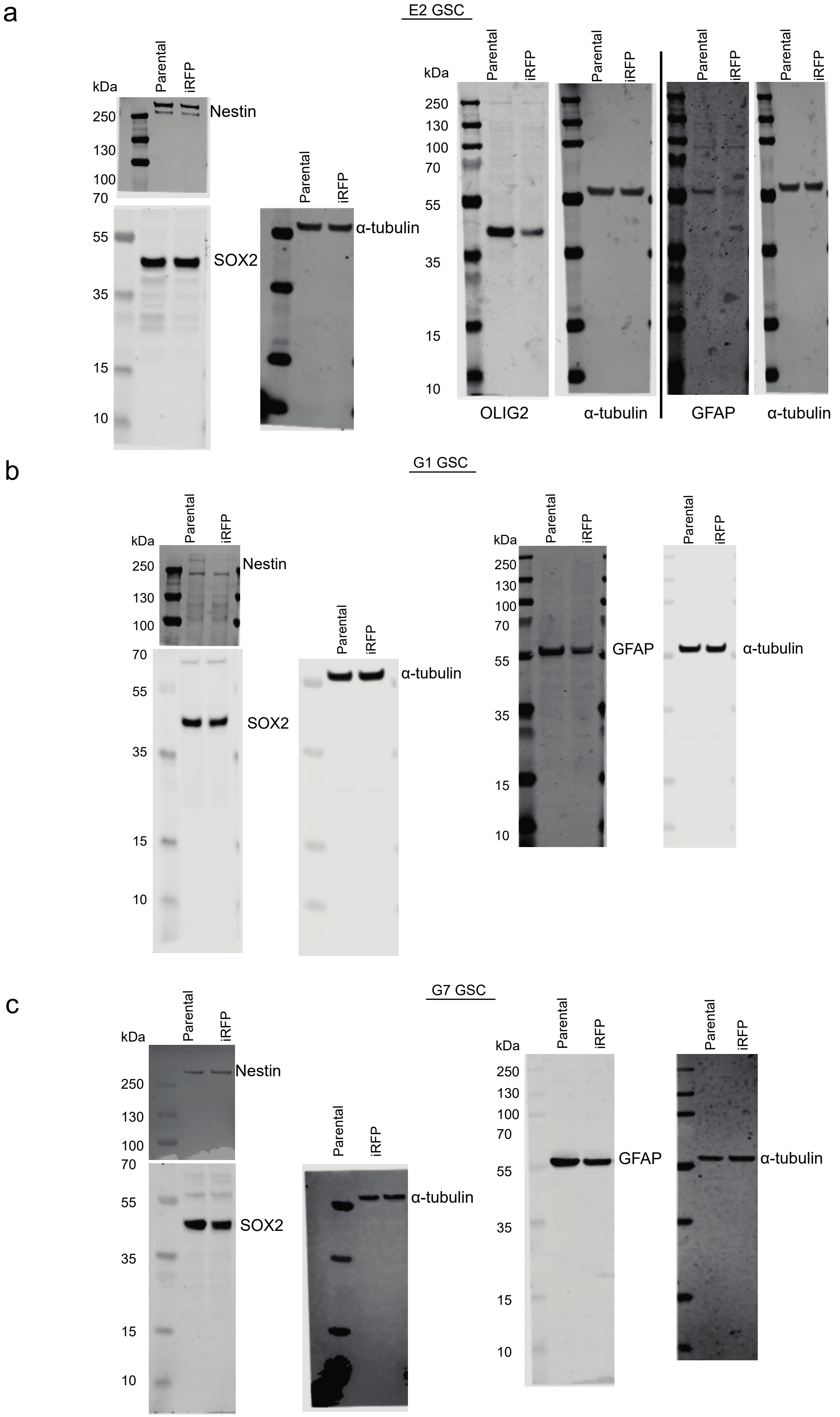

Supplemental Figure 2

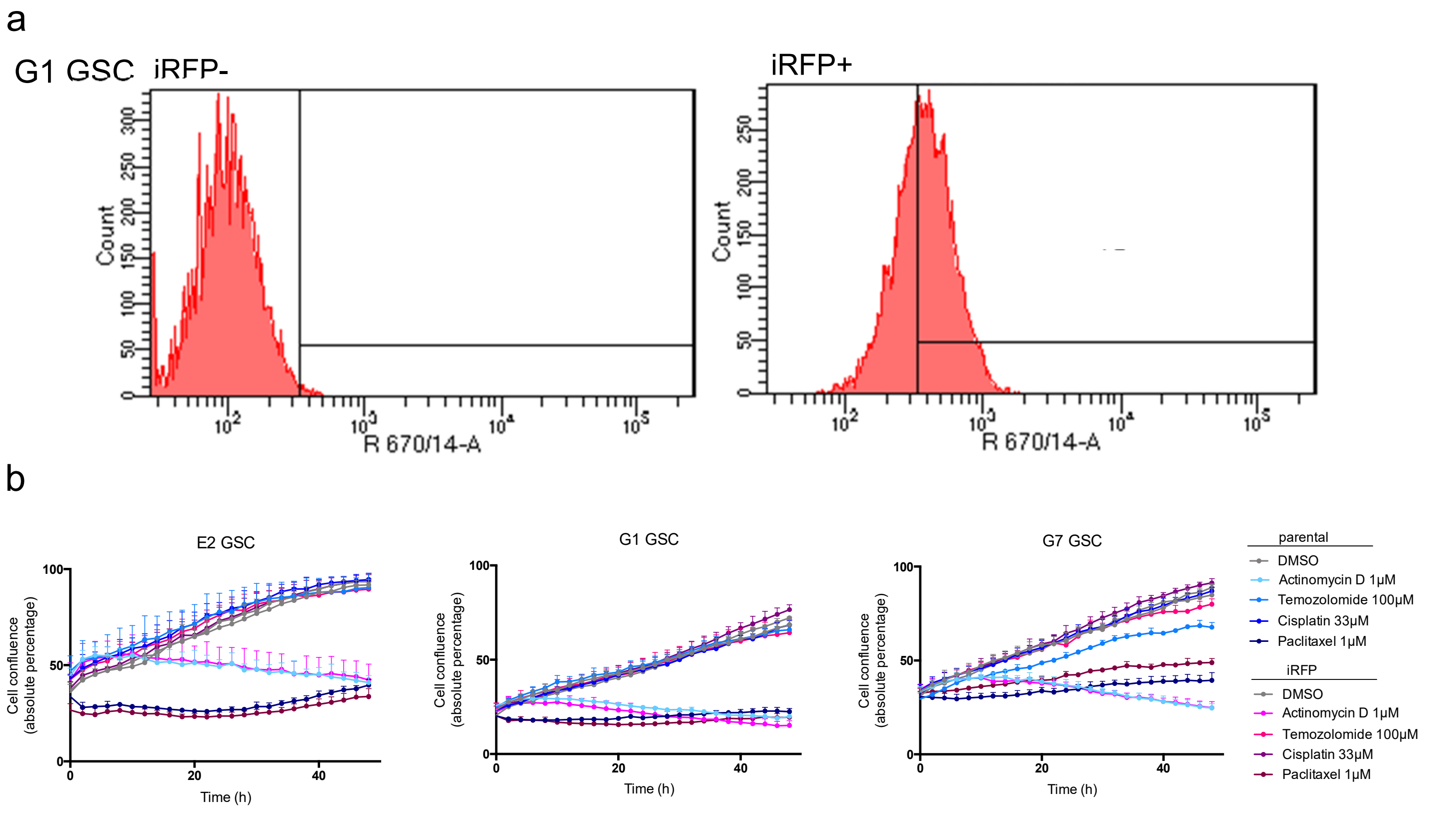

Supplemental Figure 3

a

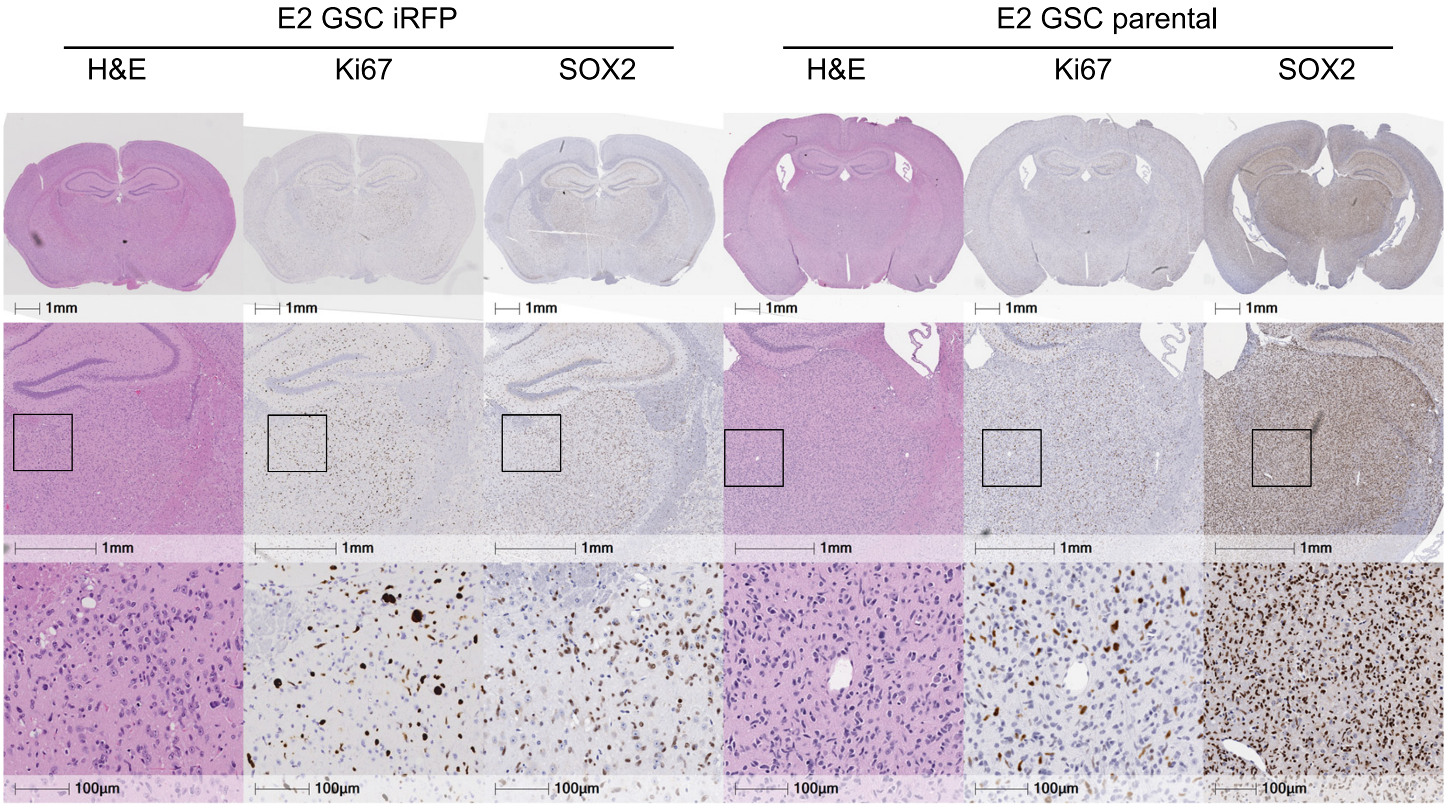

b

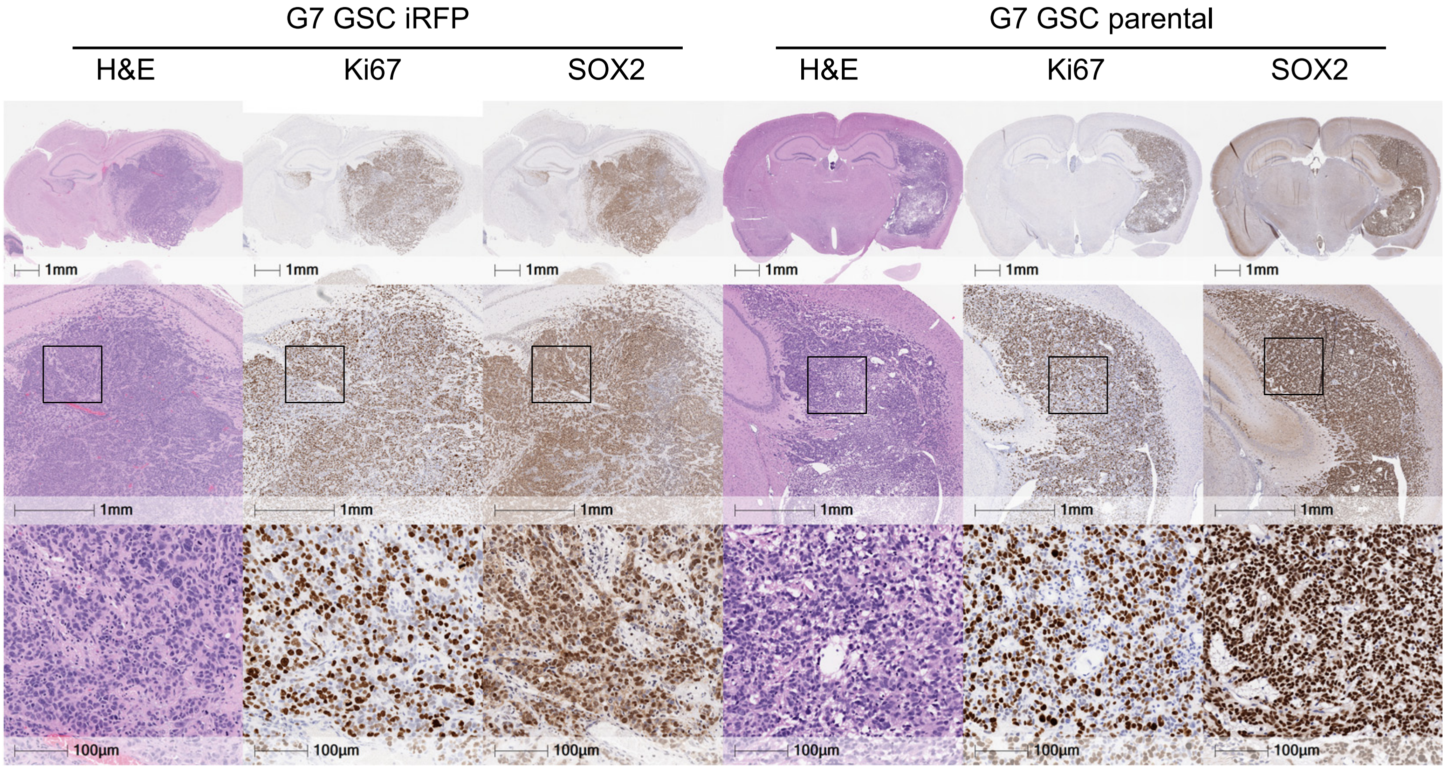

Supplemental Figure legends

Supplemental Figure 1

Full length scans of immunoblots shown in Figure 2 (a) E2 GSC (b) G1 GSC (c) G7 GSC.

Supplemental Figure 2

(a) FACS analysis of parental (iRFP-) vs. iRFP expressing (iRFP+) G1 GSC. (b) Cell confluence assay after treatment with indicated chemotherapeutic agents. Every two hours over a 48-hour time period images were taken in the IncuCyte Zoom. One representative experiment of three independent repeats is shown. Error bars represent mean +/-SEM.

Supplemental Figure 3

(a) Representative images of haematoxylin and eosin (H&E), Ki67 and SOX2 immunohistochemistry of mouse brain sections with orthotopic PDX from E2 GSC iRFP and parental (a) and G7 GSC iRFP and parental (b).
